# Advances in Multimodal Artificial Intelligence in Radiology: Data Integration, Foundation Models, and Clinical Applications—A Narrative Review

**DOI:** 10.3390/healthcare14193216

**Published:** 2026-09-29

**Authors:** Ghada Alfattni

**Affiliations:** Department of Computer Science, Jamoum University College, Umm Al-Qura University, Makkah 24382, Saudi Arabia; gafattni@uqu.edu.sa

**Keywords:** multimodal artificial intelligence, radiology, medical imaging, clinical data, vision–language models, foundation models, large language models, clinical decision support, radiology report generation, narrative review

## Abstract

**Background/Objectives:** Multimodal artificial intelligence (AI) is increasingly used in radiology to combine medical images with radiology reports, clinical narratives, structured health records, laboratory measurements, and other patient data. These systems may support more context-aware interpretation, reporting, and clinical decision-making than unimodal approaches. This narrative review examines recent technical and clinical advances in multimodal radiology AI and identifies barriers to responsible implementation. **Methods:** Relevant biomedical and technical literature was identified through targeted searches of PubMed, IEEE Xplore, ACM Digital Library, Web of Science, Scopus, arXiv, ScienceDirect, SpringerLink, and Google Scholar. A documented screening process identified 111 reviewed publications from 1154 records. Study selection and data extraction were conducted by one reviewer without independent verification. Original research, reviews, and commentaries, including selected preprints, were synthesized thematically. **Results:** The field has progressed from early and late feature fusion toward cross-modal attention, contrastive image–text pretraining, vision–language models, multimodal large language models, and general-purpose foundation models. Applications include diagnostic classification, prognostic modelling, image–text retrieval, visual question answering, clinical decision support, and automated radiology report generation. Despite promising technical results, comparison across studies remains difficult because of heterogeneous datasets, tasks, metrics, and validation designs. Clinical translation is further limited by scarce external and prospective validation, uncertain interpretability, hallucination and omission risks, privacy and fairness concerns, and limited real-world workflow evaluation. **Conclusions:** Multimodal AI may enable more clinically informed radiological interpretation and reporting, but progress in benchmark performance has outpaced evidence of safety, generalizability, and clinical utility. Future research should prioritize multicentre evaluation, clinically meaningful metrics, transparent reporting, robust safety assessment, and workflow-centred implementation.

## 1. Introduction

Artificial intelligence (AI) encompasses computational approaches to tasks such as pattern recognition, prediction, and decision support. Machine learning (ML) is a branch of AI in which models learn relationships from data. Artificial neural networks are one family of ML models, and deep learning uses networks with multiple layers to learn increasingly complex representations. These approaches are being investigated in medicine to support diagnosis and treatment using clinical data. Kufel et al. provide an overview of these concepts and examples of their medical applications [1]. This background provides a foundation for examining how radiology AI combines imaging with other clinically relevant information.

Radiology is central to disease detection, diagnosis, treatment planning, and assessment of therapeutic response. AI has supported the development of computer-assisted systems for image classification, detection, segmentation, prognosis, and reporting [2]. In clinical practice, however, radiological interpretation rarely depends on images alone. Radiologists also consider the clinical indication, patient history, prior examinations, radiology reports, laboratory measurements, and information from electronic health records; in selected applications, pathology and molecular data may also be relevant [3]. In this review, multimodal radiology AI therefore refers to systems that combine radiological images with one or more textual, structured, or biological sources of patient information.

Image-only models can identify visual patterns but cannot directly account for clinical context that is absent from the pixels [4]. Conversely, natural language processing (NLP) systems can extract information from reports and clinical narratives but cannot independently evaluate the underlying images [5]. Combining modalities may provide complementary information and align computational models more closely with clinical decision-making. The benefit is not automatic, however: additional inputs may be redundant, incomplete, temporally inappropriate, or affected by information leakage and institutional bias. The value of a multimodal design must consequently be demonstrated for the specific task and clinical setting rather than inferred from the number of modalities alone.

Technical approaches have progressed from feature concatenation and ensembles of unimodal predictions to cross-modal attention, shared representation learning, and contrastive image–text pretraining [6,7]. Vision–language models (VLMs), multimodal large language models (LLMs), and foundation models have extended these approaches to image–text retrieval, visual question answering, report generation, image grounding, diagnostic support, and outcome prediction [8,9]. Nevertheless, comparison across systems remains difficult because studies use different datasets, patient populations, modalities, tasks, outcome definitions, and evaluation metrics.

Clinical translation introduces concerns that are not captured by benchmark performance alone. These include external validity, calibration, robustness to missing or discordant inputs, interoperability, interpretability, workflow fit, privacy, and fairness [10,11]. Generative systems introduce additional risks of unsupported statements, omitted findings, and overconfident language. Although foundation models are intended to transfer across tasks, institutions, and imaging modalities, evidence of reliable generalization in real clinical settings remains limited [12]. These questions are particularly relevant as radiology services seek safe methods of managing increasing imaging volumes and reporting workloads [13].

Previous reviews provide complementary perspectives on this field. Summaira et al. survey multimodal deep learning across diverse applications and data types, including images, text, audio, and physiological signals [6]. Chen et al. examine vision–language pretraining, organizing developments around feature extraction, architectures, training objectives, datasets, and downstream tasks [8]. Khan et al. survey foundation models across medicine, encompassing clinical language processing, medical imaging, and omics [12]. Within radiology, Nensa discusses the transition toward multimodal diagnostics and its implications for radiologists’ roles, training, and interdisciplinary practice [9].

The present review brings these perspectives together around radiological applications, examining task-specific data fusion alongside vision–language pretraining, foundation models, and report generation. Its particular emphasis is the relationship between model design and evidentiary strength: whether additional modalities contribute demonstrable value, whether inputs are available at the intended clinical decision point, how performance is validated, and which limitations constrain clinical use. This provides a common basis for interpreting developments across diagnostic, prognostic, and reporting applications.

The motivation for this review is the need to connect developments in multimodal radiology AI with the evidence required to judge their clinical value. Combining imaging with other patient information creates opportunities for more context-aware prediction and reporting, but it also raises questions about whether additional inputs provide complementary information, whether they are available at the intended clinical decision point, and whether reported improvements generalize beyond the development dataset. Readers therefore need a synthesis that examines both technical capabilities and the strength of the evidence supporting their proposed use.

This narrative review examines data integration strategies, diagnostic and prognostic applications, vision–language and foundation models, and automated report generation through a common set of questions: what information is combined, what benefit is demonstrated, how that benefit is validated, and what limitations affect clinical interpretation. Its objectives are to explain major technical developments, critically compare evaluation practices, and identify priorities for clinically meaningful validation and implementation.

## 2. Review Approach and Scope

### 2.1. Review Design

This article presents a narrative synthesis of recent developments informed by a documented literature search and screening process. A narrative approach was selected because multimodal AI in radiology is rapidly evolving and encompasses heterogeneous data sources, model architectures, clinical tasks, and evaluation practices. The objective was to explain major developments, compare their clinical implications, and identify translational challenges rather than to estimate a pooled effect or provide an exhaustive inventory of publications.

The scope is organized around the integration of radiological imaging with other clinically relevant information. The topics covered represent different dimensions of that process: data fusion describes how inputs are combined; vision–language and large language models describe model capabilities and architectures; foundation models concern pretraining and reuse; and report generation and clinical decision support describe applications. These categories overlap and are not treated as mutually exclusive model classes. They are examined together to connect integration choices with their evaluation and clinical implications. The review prioritizes these relationships rather than attempting an exhaustive technical account of every architecture.

### 2.2. Information Sources and Search Approach

Relevant literature was identified through targeted searches of PubMed, IEEE Xplore, ACM Digital Library, Web of Science, Scopus, arXiv, ScienceDirect, SpringerLink, and Google Scholar. Search terms combined concepts relating to multimodal artificial intelligence, radiology, medical imaging, clinical data, radiology reports, electronic health records, vision–language models, large language models, foundation models, multimodal fusion, and report generation. Reference lists of relevant primary studies and reviews were also examined to identify additional work. The review emphasized English-language literature published from 2018 onward, while earlier publications were considered when they provided important conceptual or methodological context. The final search was conducted on 30 April 2026.

**Figure 1:**
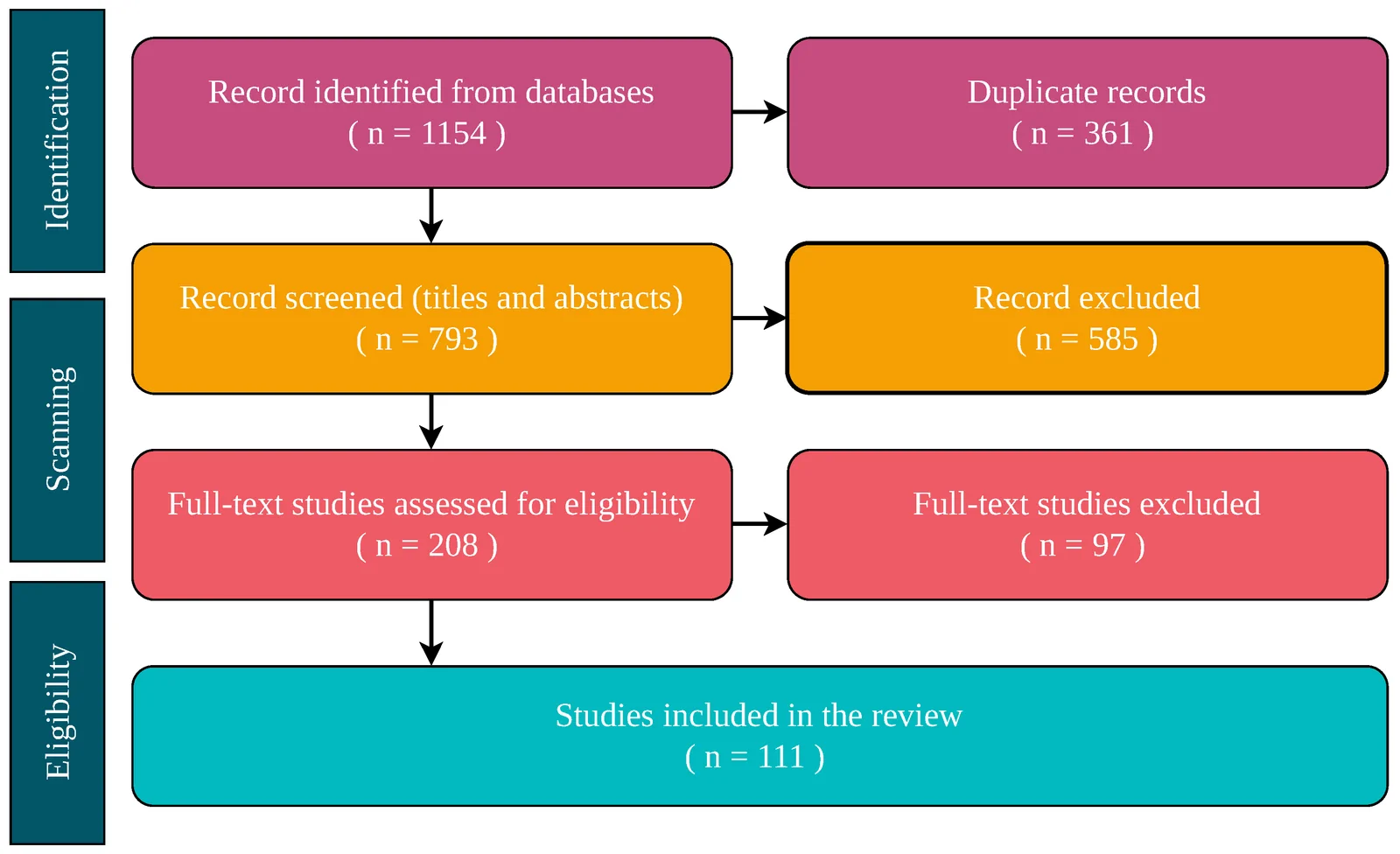
Documented publication-selection flow. The final total includes original research, reviews, and commentaries. The diagram uses an adapted presentation informed by PRISMA 2020; full-text exclusions are reported as an aggregate rather than as reason-specific counts.

The search and screening process was documented, and the selected literature was synthesized narratively. Figure 1 summarizes the selection stages using an adapted flow presentation informed by PRISMA 2020 [14]. This use of PRISMA reporting elements does not imply full compliance with the systematic-review checklist.

### 2.3. Scope and Selection Principles

The reviewed literature included original research, reviews, and commentaries. For empirical model evaluations, selection focused on research combining radiological imaging with at least one additional clinically relevant information source, such as radiology reports, clinical narratives, structured electronic health record variables, laboratory measurements, pathology, or molecular data, with quantitative evaluation using medical data. Reviews and commentaries informed the conceptual, methodological, and clinical interpretation. English-language journal articles, conference papers, selected preprints, and relevant book chapters were considered. The review emphasized publications from 2018 onward, while earlier publications were considered when relevant.

For empirical model reports, exclusions concerned single-modality investigations, absence of quantitative evaluation, exclusively synthetic or non-clinical data without validation on real patient data, insufficient methodological detail, or lack of relevance to radiological practice. These empirical criteria did not apply to reviews and commentaries, which were included for their relevance to the review’s scope. The total therefore counts reviewed publications across publication types, rather than independent primary studies.

The search identified 1154 records. Following removal of 361 duplicates, 793 records underwent title and abstract screening. Of these, 585 were excluded, and 208 articles underwent full-text assessment. A further 97 articles were excluded, leaving 111 reviewed publications in the final narrative synthesis, including original research, reviews, and commentaries. Full-text exclusions concerned insufficient methodological detail, absence of eligible multimodal integration, or limited relevance to radiology; only the aggregate exclusion count is presented here.

Study selection and data extraction were performed by a single reviewer (G.A.). No second reviewer independently verified eligibility decisions or extracted information.

Preprints were eligible to capture emerging developments in this rapidly evolving field. Their findings were interpreted with caution because they had not necessarily undergone peer review at the search date. The historical background references on radiological decision-making and clinical NLP [3,5] were excluded from the reported 111 reviewed publications. Additional contextual and methodological references introduced during revision also remain outside this total. Journal versions published after the search cutoff do not retrospectively change the preprint status of the reviewed evidence.

### 2.4. Thematic Synthesis

The literature was organized into seven themes: multimodal data fusion and integration; diagnostic and prognostic applications; LLMs and VLMs in medical imaging; automated radiology report generation; multimodal foundation models; condition-specific applications; and ethical, regulatory, and implementation considerations. For empirical studies, the synthesis considered the data modalities, model architecture, fusion strategy, clinical task, dataset, reported metrics, validation design, and stated limitations, where applicable. Reviews and commentaries contributed interpretation and context; their statements are not treated as additional independent empirical findings.

Because datasets, outcomes, metrics, and experimental designs differed substantially, results were synthesized narratively and were not pooled. Reported performance values were treated as study-specific and not as direct rankings across models. No formal risk-of-bias or certainty-of-evidence assessment was undertaken. Instead, methodological limitations—including weak comparators, absence of modality ablation, possible information leakage, limited external validation, and incomplete reporting—were considered qualitatively when interpreting the literature. The thematic tables present selected examples; Appendix B lists all 111 reviewed publications. The interpretation distinguishes findings reported in individual studies from methodological recommendations developed in this review.

## 3. Thematic Review of Recent Advances

### 3.1. Evolution of the Field

The development of multimodal AI in radiology can be understood as a progression through several overlapping technical directions rather than as a fixed sequence. Earlier task-specific systems commonly combined independently extracted image features with structured clinical variables or text representations through concatenation, joint classifiers, or ensembles. These approaches established that non-imaging information could alter predictions, but they often captured only limited interactions between modalities and depended on carefully curated inputs.

Cross-modal representation learning subsequently shifted attention from combining final features or predictions to learning relationships between image regions and clinical concepts. Global–local alignment and contrastive pretraining on paired images and reports enabled models to learn reusable representations from data generated during routine care [15,16]. Transformer-based attention further supported interactions among image regions, report sentences, structured variables, and longitudinal information. These developments expanded multimodal AI beyond individual classification tasks toward retrieval, transfer learning, grounded interpretation, and report generation.

More recent work has emphasized VLMs, multimodal LLMs, and foundation models that can support several tasks through a shared architecture. Representative systems have been applied to conversational image interpretation, visual question answering, automated reporting, image grounding, and workflow-oriented tools [17,18,19]. This direction also changes the evaluation problem: a model that generates free text or interacts with users must be assessed for factuality, omissions, uncertainty, grounding, and usability in addition to conventional classification performance.

Technical expansion should not be equated with clinical maturity. Much of the literature remains retrospective and benchmark-oriented, frequently relying on public datasets or data from a limited number of institutions. External validation, prospective evaluation, reader studies, and assessments of workflow or patient outcomes remain less developed than model architecture and pretraining. The following sections therefore consider both technical advances and the strength of evidence supporting their use in radiological practice.

### 3.2. Multimodal Data Fusion and Integration

Multimodal systems differ in where and how they combine information. In early fusion, raw inputs or independently extracted features are concatenated before prediction. This design is comparatively simple and has been used to combine chest radiographs or CT with clinical text, electronic health record variables, or laboratory measurements [20,21,22]. Its limitations include sensitivity to feature scaling, missing inputs, and temporal misalignment between the imaging examination and the associated clinical data.

Intermediate fusion allows modalities to interact within the model through shared latent representations, cross-modal attention, co-attention, or transformer layers. Such methods can model conditional relationships—for example, when the relevance of an image region depends on the clinical history—and have been investigated for chest pathology classification, disease analysis, and survival prediction [23,24,25]. Attention maps may help inspect these interactions, but they should not by themselves be interpreted as causal or clinically valid explanations.

Late fusion combines predictions from separately trained unimodal models. Although it captures fewer fine-grained interactions, it is modular, easier to audit, and can sometimes tolerate an unavailable modality [26]. A further family of methods uses large-scale image–text pretraining rather than task-specific fusion. BioMedCLIP and GLORIA, for example, learn aligned visual and textual representations that can be transferred to retrieval, classification, and other downstream tasks [15,16]. Multidomain systems may additionally combine radiology, pathology, and molecular data, as illustrated in non-small-cell lung cancer [27].

The fusion strategies in Table 1 address different integration problems, and their relative value depends on the clinical task and available inputs. Early fusion offers a straightforward way to combine features, but its usefulness depends on consistent preprocessing and alignment. Intermediate fusion enables interactions between modalities, although architectural complexity alone does not demonstrate that these interactions improve prediction. Late fusion provides modularity, but combining predictions may overlook relationships that require joint interpretation. Image–text pretraining addresses a related but distinct objective: learning transferable representations rather than necessarily combining patient-specific inputs during clinical inference.

These distinctions affect how evidence should be interpreted. Improvement over an image-only baseline does not, by itself, establish that a particular fusion mechanism is responsible for the gain. A convincing comparison requires image-only, non-imaging-only, and multimodal baselines evaluated using the same patients, outcomes, and data partitions. Modality-ablation experiments should establish whether each input contributes useful information, while comparisons with simpler fusion methods should test whether additional complexity is justified.

Clinical relevance also depends on when the information becomes available. A report used as supervision during pretraining has a different role from clinical history supplied during inference. Using a final diagnostic report as an input to predict the diagnosis from the same examination can introduce target leakage. Evaluation should therefore document input timing, patient-level data separation, and performance when modalities are missing or discordant. Without these checks, reported gains provide limited evidence that a fusion strategy will remain useful in the intended clinical workflow.

### 3.3. Diagnostic and Prognostic Applications

Diagnostic and prognostic studies have combined imaging with clinical history, laboratory measurements, physiological signals, pathology, and molecular data. In thoracic applications, these inputs have been used for lung-cancer classification, pulmonary-infection assessment, and pulmonary-embolism detection [22,30,31]. The authors of several studies reported gains over selected unimodal baselines, but the size and reliability of those gains cannot be compared directly because the cohorts, outcomes, and validation procedures differed.

Oncology provides broad examples of data integration. Investigated combinations include CT with clinical follow-up data in renal-cell carcinoma, MRI-derived tumour habitats with clinicopathological variables in ovarian cancer, and radiology–pathology–genomic data in non-small-cell lung cancer [27,32,33]. These studies illustrate possible roles in diagnosis, recurrence prediction, and treatment-response assessment. They also introduce risks of small effective sample sizes, incomplete molecular data, and overfitting when the number of features is large relative to the number of patients.

Other applications use temporal or contextual information that cannot be inferred from a single image. Cardiovascular literature describes the integration of multiple imaging modalities with clinical information, while an EHR-based risk-prediction study combined physiological time series, chest radiographs, and clinical notes [34,35]. Neurological models have aligned MRI with radiology reports, clinical narratives, or tumour-location information for brain-disease and paediatric brain-tumour applications [36,37,38]. Interpretability claims in these studies require caution: feature attribution or attention visualization can indicate model sensitivity but does not establish that the model used clinically valid reasoning.

Table 2 is intentionally qualitative: its studies address different outcomes and populations. Across these applications, the key question is whether additional modalities improve a clinically relevant prediction beyond what is achievable with imaging or clinical information alone. The presence of several input types does not establish their individual contribution. Comparisons should therefore include both imaging-only and clinical-only baselines under the same evaluation conditions. Otherwise, an apparent multimodal benefit may reflect the predictive strength of one input rather than complementary information learned across modalities.

Diagnostic and prognostic tasks also require different interpretations of input timing. For diagnostic support, each input should be available when the proposed decision is made. For prognosis, predictors must precede the outcome and correspond to a clearly defined prediction time. Follow-up information may legitimately define an outcome, but using information acquired after the prediction time as a predictor would compromise the interpretation of performance. These distinctions are particularly important when imaging is combined with longitudinal clinical records, pathology, or molecular measurements.

Validation design determines the strength of the resulting evidence. Internal testing assesses performance within the development setting, whereas external testing examines transfer to a different population or institution. Neither alone demonstrates that using the model improves clinical decisions. For risk prediction, discrimination should be considered alongside calibration and performance at clinically relevant decision thresholds. For diagnostic applications, sensitivity and specificity should be interpreted in relation to the intended role and the consequences of missed findings or false alerts.

The studies summarized here therefore illustrate potential uses of multimodal information rather than establish a general advantage across diseases. Stronger conclusions require study-specific evidence of incremental benefit, appropriate input timing, and validation that matches the proposed clinical use.

Table 3 provides within-study quantitative comparisons for two representative predictive applications. All values are reported AUROCs; differences across studies should not be interpreted as model rankings. Separate rows distinguish internal and external testing in the pulmonary-infection study.

The reported point estimates suggest an absolute AUROC increase of 0.10 over the CT-only DyAM comparator in Vanguri et al. and increases of 0.016 internally and 0.031 externally over the image model in Shao et al. These calculated differences describe the reported comparisons; they are not pooled effects, estimates of patient benefit, or evidence that either integration strategy is superior across applications. In particular, the small external cohort in Shao et al. and the lack of an external integrated-model cohort in Vanguri et al. constrain different aspects of generalizability.

### 3.4. LLMs and VLMs in Medical Imaging

LLMs and VLMs have related but distinct roles in radiology. Text-only LLMs can summarize, restructure, or retrieve information from clinical language, whereas VLMs and multimodal LLMs accept visual and textual inputs and can produce classifications, free-text responses, image–text matches, or grounded descriptions. The literature represented in Table 4 spans general radiology, chest radiography, specialized imaging, technical adaptation, evaluation, and workflow-oriented interfaces. Most of these systems remain research prototypes, and benchmark capability should not be interpreted as readiness for autonomous clinical use.

Chest radiography is a prominent test domain, supported by large paired image–report datasets. Systems such as CXR-LLaVA and M4CXR investigate conversational interpretation, diagnostic classification, and report generation [42,43]. More specialized work includes evaluation of multimodal models for brain-tumour MRI reasoning, three-dimensional brain-CT report generation, and ultrasound documentation [45,46,47]. This breadth demonstrates technical adaptability, but evidence is uneven across modalities; success on two-dimensional chest radiographs may not transfer to volumetric, temporal, or operator-dependent imaging.

Current development also includes parameter-efficient fine-tuning, segmentation-oriented reasoning, reinforcement learning, and image grounding [48,49,50,56]. These methods attempt to adapt large models with fewer trainable parameters or to link generated language to image regions. Grounding may make an output easier to inspect, but it does not by itself guarantee diagnostic correctness or exclude reliance on spurious visual features.

Agentic systems and chatbot interfaces extend the model from a single prediction to a sequence of tool calls or interactions [19,44,51,52]. Such systems could support case preparation, retrieval, measurements, or report drafting; they also create additional failure points because an early error may propagate through later steps. Claims that an agent reproduces radiologist reasoning should consequently be supported by prospective workflow studies rather than by text similarity or benchmark accuracy alone.

The systems discussed in this section should be interpreted according to their training objectives, inference inputs, and outputs. Image–text retrieval, diagnostic classification, visual question answering, and free-text reporting test different capabilities. Strong retrieval performance demonstrates useful alignment between images and text, but does not establish that a model can generate clinically accurate reports. Similarly, performance on constrained questions provides limited evidence about the reliability of unrestricted responses.

The contribution of imaging requires particular scrutiny in question-answering systems. A model may produce plausible answers from linguistic patterns or clinical context without adequately using the image. Comparisons with text-only inputs, removal of imaging information, and testing with mismatched image–text pairs can help assess whether responses depend on relevant visual evidence. These analyses complement conventional accuracy measures by examining whether the system uses the information required for its proposed role.

Grounding and factual correctness also require separate assessment. Linking a statement to an image region can make an output easier to inspect, but does not establish that the statement is correct or that other important findings have been captured. Evaluation should distinguish finding recognition, anatomical localization, unsupported statements, and omissions. It should also specify whether clinical history or prior examinations were available to the model.

The clinical significance of these systems therefore depends on more than the breadth of tasks they can perform. Evidence should establish which capabilities remain reliable under external testing, how failures are detected, and whether users can verify outputs efficiently. These distinctions provide a basis for judging whether a model’s demonstrated capabilities support its proposed clinical application.

### 3.5. Automated Radiology Report Generation

Automated report generation maps imaging information to free-text findings or impressions. Proposed uses include drafting, standardizing terminology, and reducing repetitive documentation; these systems do not reproduce the full cognitive process of a radiologist and should be evaluated as decision-support or drafting tools. Development has moved from templates and recurrent language models toward transformer-based image encoders, volumetric attention, multimodal pretraining, and grounded generation.

Representative systems illustrate different technical directions. XRaySwinGen combines a hierarchical vision transformer with a language decoder for chest-radiograph reporting [57]. CT2Rep addresses volumetric CT, where information must be integrated across many slices rather than a small number of projection images [58]. MAIRA-2 produces image-grounded reports that associate generated statements with image regions [17], while UIT-2Q2T examines bootstrapped image–language pretraining [59]. These contributions are summarized qualitatively in Table 5; their reported scores are not presented as a ranking because they were obtained with different datasets, targets, and evaluation protocols.

Language and institutional transfer are additional concerns. Adaptation to settings with limited domain-specific text requires more than translation because terminology, report structure, and diagnostic conventions vary across institutions and languages [60]. External testing should therefore include local reporting practices and should document whether the model received clinical history, prior reports, or other information unavailable from the current image.

Evaluation must distinguish linguistic similarity from clinical correctness. In a study comparing automated metrics with radiologists’ assessments, Yu et al. found that RadGraph F1 and the composite RadCliQ metric provided useful alignment with expert judgments. Their analysis also identified differences in how metrics captured particular errors, demonstrating that a favourable aggregate score can conceal clinically relevant weaknesses. However, the evaluation concerned chest radiographs and included variability between radiologist assessments; its findings should not be assumed to transfer unchanged to CT, MRI, or other reporting settings [61].

Different model developments also require different evidence. MAIRA-2 extends chest-radiograph reporting by linking findings to image regions and incorporating reporting context. Its RadFact framework evaluates sentence-level correctness and completeness and supports assessment of spatial grounding [17]. These features make aspects of report quality more inspectable, but grounding should be assessed separately from factual accuracy and omission of findings. Because RadFact uses an LLM-based evaluator, agreement with expert assessment remains important when interpreting its scores.

CT2Rep addresses volumetric chest CT and includes a longitudinal extension incorporating information from previous examinations [58]. Its contribution therefore differs from systems designed for projection radiographs: it addresses volumetric representation and temporal context. Evaluation should establish whether generated statements accurately describe the current examination and whether statements about change are supported by the available prior information. These task differences prevent straightforward numerical ranking against chest-radiograph reporting systems.

Together, these developments support a layered evaluation approach. Text-based metrics can assess similarity to reference reports; clinically informed metrics can assess findings and relationships; and expert review can assess error severity, omissions, and consistency with the images and clinical context. Reference-report agreement alone is insufficient to establish image-grounded correctness. Comparisons should also specify whether systems generate findings, impressions, or complete reports and whether they receive clinical history or prior examinations.

Clinical utility requires an additional level of assessment. Prospective reader and workflow studies should measure the time needed to review and correct drafts, clinically important errors remaining after review, and the influence of generated text on radiologists’ decisions. Faster generation should not be equated with faster or safer reporting. A useful system must reduce the overall reporting burden while preserving the accuracy and completeness of the final report.

### 3.6. Multimodal Foundation Models in Radiology

Foundation models are pretrained on broad datasets and then adapted or prompted for multiple downstream tasks. In radiology, “foundation model” may refer to an image encoder, an image–text representation model, a generative VLM, or a generalist multimodal system. These categories should be distinguished because they differ in inputs, outputs, and validation requirements.

Generalist systems such as Lingshu, MedVersa, and Citrus-V investigate shared architectures for image–text understanding, task transfer, and image grounding [18,62,63]. Other work is domain-focused. HONeYBEE provides modality-specific pipelines and foundation-model embeddings for radiological, pathological, clinical, and molecular oncology data; a subsequent commentary discusses its translational implications [64,65]. Three-dimensional foundation-model research has also introduced paired chest-CT and report data and models for CT representation learning and question answering [66]. Generative and multi-disease frameworks explore conditional synthesis and transfer across tasks or anatomical areas [67,68]. Table 6 groups these representative directions without implying equivalence among their evaluations.

Foundation models should be evaluated according to the transfer they demonstrate. Performance across several tasks on a familiar dataset, transfer to a new institution, and adaptation to a different imaging modality represent distinct achievements. A model’s scale or range of supported tasks does not establish that it will generalize across these settings. Evaluation should specify which components were pretrained, what adaptation was required, and whether downstream test data were independent of the pretraining sources.

The role of each modality also requires clarification. Image–text pretraining may use reports to supervise representation learning even when the downstream system receives only images. This differs from a system that requires patient-specific text, laboratory measurements, or other inputs during inference. The distinction affects both the interpretation of multimodal benefit and the practical requirements for deployment.

A useful assessment should therefore consider transfer performance alongside adaptation costs, input availability, and robustness. Comparisons with task-specific models should use equivalent evaluation conditions and report the amount of labelled data and fine-tuning required. Testing should also examine missing inputs and shifts in patient populations, acquisition protocols, and reporting practices.

These considerations place limits on claims of general-purpose clinical capability. Evidence of transfer on selected benchmarks supports further investigation, but broader clinical use requires validation for each intended application. The relevant question is whether reuse produces reliable performance with an acceptable adaptation and verification burden.

### 3.7. Condition-Specific Applications

Condition-specific studies illustrate how the useful combination of modalities depends on the clinical problem. A large retrospective pulmonary-infection study combined CT, clinical records, and laboratory measurements and included external testing, whereas a more preliminary tuberculosis preprint paired radiographs with clinical history [31,69]. In these settings, the central methodological question is whether non-imaging inputs add independent information available at the intended time of diagnosis, rather than merely restating the reference diagnosis.

Oncology studies have combined radiological imaging with clinicopathological or molecular information for treatment-response modelling [27,33]. An abdominal application combined CT with routine laboratory measurements, and optionally report text, for ischaemia assessment in small-bowel obstruction [70]. Table 7 summarizes these examples as a map of applications, not as comparative evidence of effectiveness.

The condition-specific applications summarized here illustrate why multimodal benefit must be interpreted within a defined clinical pathway. An additional modality is useful only if it contributes information relevant to the target decision and is available when that decision is made. Pathology or molecular measurements may support treatment-response prediction after diagnosis, for example, while being unavailable for an earlier diagnostic task. Studies using different combinations of inputs may therefore address different clinical questions even when they concern the same disease.

Access to complete multimodal data also affects the population represented in an analysis. Patients with imaging, molecular testing, and detailed follow-up may differ from those receiving routine or less extensive assessment. Evaluation should clarify how patients with missing modalities were handled and whether performance applies beyond the subset with complete records. This is particularly relevant when proposed deployment settings cannot routinely provide every required input.

The examples should consequently be interpreted as evidence for specific applications rather than a general endorsement of multimodal modelling across diseases. Their clinical significance depends on the comparator, prediction time, patient selection, and validation setting. A useful synthesis asks whether integration improves the intended decision, which patients are represented by that evidence, and what additional testing is needed before broader use.

### 3.8. Clinical Translation, Ethics, and Governance

Multimodal AI expands the amount and variety of patient information processed by one system. Linking images with free text, structured records, pathology, or genomic data can increase privacy and governance complexity because each source may have different consent, access, retention, and secondary-use conditions [10,71]. Data minimization remains important: a model should receive only information justified by its intended clinical purpose. De-identification is also more difficult when combinations of dates, rare conditions, narrative text, and images permit re-identification.

Bias can enter through every modality and through the pattern of missing data. Imaging protocols, scanner vendors, report styles, access to laboratory or molecular testing, and documentation practices vary across institutions and patient groups. A multimodal model may therefore learn institutional shortcuts even when each input seems clinically relevant. Evaluation should report subgroup performance, missingness, and the effect of withholding individual modalities, with external testing in settings that differ from the development site [11].

Generative systems require safety assessment at the level of individual claims. A fluent report or answer may contain unsupported findings, omit urgent abnormalities, misstate uncertainty, or allow clinical history to override image evidence [54]. Human oversight is necessary, but it is not sufficient unless interfaces make AI provenance and uncertainty visible and allow efficient verification. Reader and workflow studies should examine automation bias, alert fatigue, editing burden, and whether errors are detected before they reach the patient record.

Regulatory evaluation should be tied to a clearly defined intended use, user, input set, and output. A retrieval tool, report-drafting assistant, triage classifier, and autonomous diagnostic system present different risks and should not be evaluated under a single generic claim of multimodal capability. Model updates, changing input availability, and dependence on external foundation-model providers also require version control, audit trails, monitoring, and procedures for responding to performance drift.

The evidence reviewed here contains far more retrospective technical evaluations than prospective implementation studies. Responsible translation will require transparent data provenance, clinically meaningful comparators, human-factors testing, security and privacy safeguards, external validation, and post-deployment surveillance. These requirements are not obstacles separate from model development; they are part of establishing whether a multimodal system is clinically useful and safe.

Table 8 contrasts selected evaluations by the claims they support. It is an interpretive comparison, not a formal risk-of-bias assessment or ranking of models. In particular, validating a component of a multimodal system is distinct from validating the integrated model, and comparison with clinician performance is distinct from demonstrating benefit when clinicians use the system.

These examples qualify the general concern about limited validation. Vanguri et al. demonstrate a within-cohort advantage of integration, but the additional unimodal cohorts should not be mistaken for independent validation of the complete multimodal model. Shao et al. provide external testing, so their evidence should not be described simply as lacking external validation. Nevertheless, external predictive performance and comparison with physicians answer different questions from whether AI assistance improves decisions. MAIRA-2 addresses a different output and evaluation problem: factual and spatial assessment of generated reports. The common translational gap is therefore not identical across studies; it depends on which part of the proposed clinical claim remains untested.

### 3.9. Explainability in Multimodal Radiology AI

Explainable AI (XAI) for multimodal radiology must address the contribution of each input and the interactions between inputs. An image heatmap alone cannot establish whether a prediction was driven by anatomy, a diagnostic phrase, or structured clinical information. The following approaches provide a framework for examining these questions. The cited methods include general machine-learning contributions and imaging-specific evaluation; their proposed application to combined radiological inputs should not be interpreted as established clinical effectiveness.

#### 3.9.1. Modality Attribution and Interactions

Modality attribution examines how imaging, text, and structured variables influence a particular output. DIME separates unimodal contributions from multimodal interactions, providing a methodological example of why explaining each input independently may miss the behaviour of a fused model [72]. In radiology, such analysis could test whether a suspected finding depends on image evidence or predominantly on the clinical history. Removing an input from a fixed model measures sensitivity to that removal; retraining without the input evaluates a different question about incremental predictive value. Neither establishes a causal clinical effect. Replacement values and missing-input handling must be specified because implausible perturbations can create cases outside the training distribution.

#### 3.9.2. Cross-Modal Saliency and Grounding

Cross-modal saliency seeks to connect influential image regions with words or clinical variables. Grounding a report statement to an image region provides an inspectable correspondence, but does not by itself establish that the model relied on that region. Arun et al. found that none of eight saliency methods satisfied all tested trustworthiness criteria across two chest-radiograph datasets [73]. This finding concerns the evaluated image models rather than all multimodal systems. For multimodal applications, evaluation should combine annotated localization with tests of output sensitivity, model randomization, and stability under clinically equivalent wording. Laterality, negation, and conflicting history are particularly relevant challenge cases for checking whether image–text alignment reflects the intended finding.

#### 3.9.3. Concept-Based Explanations

Concept-based methods express model behaviour using clinically interpretable concepts rather than individual pixels. Testing with Concept Activation Vectors (TCAV) measures prediction sensitivity to user-defined concepts [74]. A multimodal adaptation could examine a concept such as pleural effusion separately in image and text representations and then assess its role after fusion. Concepts require independent annotation and evaluation on held-out data; agreement with diagnostic vocabulary is insufficient if the same vocabulary leaks the target label. Correlated concepts and institution-specific documentation can also confound interpretation. Concept sensitivity should therefore be treated as a model property, not proof of clinically correct reasoning.

#### 3.9.4. Counterfactual Explanations

Counterfactual explanations identify hypothetical input changes associated with a different model prediction. Diverse counterfactual methods illustrate the need to consider more than one alternative and to constrain feasibility [75]. For multimodal radiology, changing a laboratory value while retaining an incompatible history or image can produce a mathematically valid but clinically incoherent example. Proposed explanations should preserve patient identity, temporal consistency, plausible anatomy, and relationships between modalities. Synthetic image changes also require review for unintended alterations. Such examples probe the model’s decision boundary; they do not predict the effect of treatment or establish what would happen to the patient.

#### 3.9.5. Explanations That Incorporate Uncertainty

Uncertainty information can complement an explanation by indicating when its associated prediction is unreliable. Deep ensembles provide one established method for estimating predictive uncertainty in classification and regression [76]; they are not explanations in themselves. A multimodal interface could present source availability, disagreement between inputs, and calibrated predictive uncertainty alongside attribution. These quantities should remain distinguishable: a missing modality does not necessarily imply high uncertainty, and model disagreement does not identify its clinical cause. Calibration and the performance of any abstention rule require external and subgroup evaluation. For generated reports, fluent wording or high token probability should not be treated as confidence in clinical correctness.

Across these approaches, explanation quality should be evaluated separately from predictive accuracy. Technical fidelity and stability are necessary assessment targets, while clinical usefulness requires testing whether explanations improve error detection and appropriate reliance without adding excessive verification effort. Comparisons of clinicians using the same system with and without explanations can help isolate this effect. The staged evaluation proposed in Section 4.2 connects these assessments with broader evidence of clinical utility.

## 4. Discussion

### 4.1. Principal Interpretation

The central issue emerging from this review is the distinction between technical capability and evidence supporting clinical use. Multimodal systems can combine complementary information, but the significance of a reported improvement depends on the comparison performed, the timing of the inputs, and the validation setting. The evidence should therefore be interpreted at the level of the intended clinical application rather than through a general assumption that more modalities produce better decisions.

Three questions connect the otherwise heterogeneous literature. First, does each additional modality contribute information beyond the strongest relevant unimodal baseline? Second, does the demonstrated benefit persist outside the development setting? Third, does using the system improve the clinical task after accounting for errors, verification effort, and workflow requirements? These questions distinguish evidence of incremental predictive value, generalizability, and clinical utility. Success at one level does not establish success at the others.

This distinction is particularly consequential for generative systems. A reporting assistant may produce fluent and largely accurate text while still requiring substantial checking for omissions or unsupported statements. Its practical value depends on the quality of the final report and the total effort required to produce it. Likewise, a diagnostic or prognostic model may discriminate between outcomes without demonstrating that its predictions improve decisions at clinically relevant thresholds.

The review therefore supports an application-specific approach to translation. Model selection and evaluation should begin with the intended user, decision, available inputs, and consequences of error. Technical advances become clinically persuasive when their benefits remain demonstrable under these conditions.

### 4.2. Research Priorities

On the basis of this synthesis, six priorities should guide future research:

1. Define every modality, its source, and its availability at the intended clinical time point.
2. Compare image-only, non-imaging, and multimodal models under identical conditions and assess information leakage.
3. Conduct independent external validation followed, where appropriate, by prospective reader and workflow studies.
4. Use clinically meaningful measures of factuality, calibration, uncertainty, robustness, and error severity.
5. Report subgroup performance, missing-data behaviour, privacy safeguards, and data provenance.
6. Establish model-version control, human-factors evidence, post-deployment monitoring, and clear responsibility for failures.

Table 9 translates these priorities into a proposed staged evaluation pathway. It is an interpretive framework derived from the synthesis, rather than a validated scoring system or a universal regulatory sequence. Before evaluation, investigators should specify the intended user, clinical decision, input availability, comparator, model version, and clinically acceptable error limits. Progression should depend on prespecified, task-specific criteria rather than improvement in a single benchmark metric.

The reporting of early live clinical evaluations can be supported by DECIDE-AI, while randomized impact trials can use the CONSORT-AI extension with the applicable core trial-reporting guidance [77,78]. These guidelines support transparent reporting; adherence alone does not establish methodological quality or clinical benefit. For report-generation systems, prospective endpoints should include consequential errors in the final signed report and total verification effort. For predictive systems, endpoints should reflect the intended decision and, where relevant, downstream patient outcomes. Failure at any stage should prompt investigation and appropriate re-evaluation rather than automatic progression.

### 4.3. Limitations of This Review

Although the review used a documented search and screening process, study selection and data extraction were performed by a single reviewer without independent verification. This may have increased the risk of selection bias, missed eligible studies, and extraction errors. The English-language restriction may also have introduced language and geographic bias. Inclusion of preprints enabled coverage of emerging developments but introduced evidence that had not necessarily undergone peer review at the search date. No formal risk-of-bias or certainty-of-evidence assessment was undertaken, and the synthesis relied on information reported in the source publications.

The 111 reviewed publications include reviews and commentaries as well as original research; this total is not a count of independent empirical evaluations. Secondary discussion may overlap with primary reports and does not provide additional confirmation of clinical effectiveness. The literature was also heterogeneous in clinical tasks, datasets, model architectures, outcomes, metrics, and validation designs, so pooled estimates and unadjusted between-study rankings were not appropriate. The selected quantitative table presents within-study contrasts, without estimating an overall effect of multimodal integration. Thematic tables present selected examples; Appendix B provides the complete publication inventory. The synthesis does not establish the prevalence of particular architectures or comparative clinical effectiveness, and absence from the discussion should not be interpreted as absence from the field. Relevant work may also have appeared after the final search on 30 April 2026.

The review describes recent technical developments rather than establishing a definitive ranking of the best-performing or most clinically mature systems. Selected preprints were included to capture emerging approaches, but their findings warrant cautious interpretation. Publication status was considered descriptively and was not used as a substitute for methodological appraisal. Because no formal assessment of risk of bias or certainty of evidence was undertaken, the synthesis cannot establish the reliability of the evidence as a whole or support strong comparative-effectiveness conclusions.

## 5. Conclusions

Multimodal AI is broadening radiology AI from isolated image analysis toward systems that combine imaging with text, structured clinical information, laboratory data, pathology, or molecular data. The reviewed literature demonstrates technical feasibility across prediction, retrieval, report generation, grounding, and interactive applications. In selected studies, multimodal systems improved specified outcomes relative to unimodal comparators. These findings do not establish universal superiority or readiness for autonomous clinical use.

The next phase of the field requires stronger evidence rather than model scale alone. Future studies should justify the timing and contribution of every modality, use unimodal and clinical comparators, assess information leakage, and conduct independent external validation followed by prospective reader and workflow evaluation where appropriate. Generative and foundation-model systems require particular attention to factuality, omissions, calibration, traceability, human oversight, model updates, and post-deployment monitoring. With these safeguards, multimodal AI may become useful decision support in radiology; until then, benchmark improvements should be interpreted as research findings rather than evidence of clinical effectiveness.

## Figures and Tables

**Table 1:** Representative multimodal fusion strategies in radiology AI.

Strategy	Integration Approach	Practical Considerations	Representative Sources
Early fusion	Concatenation of inputs or extracted features before prediction	Simple implementation; requires aligned, consistently available inputs	[20,21,22]
Intermediate fusion	Cross-modal attention, hierarchical transformers, or shared latent representations	Models interactions between modalities; interpretation of attention remains uncertain	[23,24,25]
Late fusion	Combination of predictions from unimodal models	Modular and auditable; may miss fine-grained cross-modal relationships	[26]
Image–text pretraining	Contrastive or global–local alignment of paired images and text	Supports transfer and retrieval; performance depends on data scale, quality, and domain match	[15,16,28]
Multidomain fusion	Joint modelling of radiology, pathology, clinical, or molecular data	May support precision medicine; increases missing-data and harmonization challenges	[27,29]

**Table 2:** Representative diagnostic and prognostic applications of multimodal AI.

Clinical Area	Example Modalities	Investigated Tasks	Representative Sources
Thoracic disease	CT or radiography; clinical variables; reports; laboratory tests	Cancer classification, infection assessment, and embolism detection	[22,30,31]
Gynaecological cancer	MRI; clinicopathological variables	Treatment-response prediction	[33]
Renal-cell carcinoma	CT/MRI radiomics; pathology; clinical or molecular data	Recurrence prediction	[32]
Cardiovascular and acute-care risk	Cardiovascular imaging or chest radiography; notes; physiological variables	Risk stratification and outcome prediction	[34,35]
Neurological disease	MRI; radiology reports; clinical narratives; tumour location	Differential diagnosis, disease classification, and tumour characterization	[36,37,38]

**Table 3:** Quantitative comparisons of unimodal and multimodal prediction in selected studies. Parentheses indicate reported 95% confidence intervals.

Study and Task	Dataset and Patient Counts	Modalities	Unimodal Baseline AUROC	Multimodal AUROC	External Validation
Vanguri et al.: NSCLC immunotherapy response [27]	MSK Cancer Center: 247-patient multimodal cohort; tenfold cross-validation.	CT, PD-L1 pathology, genomics.	CT-only DyAM: 0.70 (0.62–0.78).	Three modalities with PD-L1 TPS: 0.80 (0.74–0.86).	No external cohort for the integrated model.
Shao et al.: four-class pulmonary infection, internal test [31]	WCH: training 19,046; validation 2432; internal test 2433.	CT and clinical records, including laboratory data.	Image: 0.894 (0.890–0.900); clinical: 0.850 (0.844–0.856).	0.910 (0.904–0.916).	Internal test row; external results below.
Shao et al.: same task, external test [31]	CSJH: external test 196; overall primary cohort across sites 24,107.	Same input categories.	Image: 0.856 (0.840–0.872); clinical: 0.840 (0.824–0.860).	0.887 (0.867–0.909).	Yes: held-out second institution.

MSK: Memorial Sloan Kettering Cancer Center; WCH: West China Hospital; CSJH: Chengdu ShangJin Nanfu Hospital; TPS: tumour proportion score. For Vanguri et al., 247 is the overall cohort size, not the number with every modality available: delineable CT lesions were available for 187 patients and qualifying PD-L1 slides for 201. The CT-only DyAM comparator is used instead of the weaker lesion-averaging baseline. Additional radiology (n=50) and pathology (n=52) cohorts evaluated unimodal components internally and do not constitute external validation of the integrated model. For Shao et al., the numerical comparisons are taken from the primary article’s Table 1 and patient counts from Figure 1. No confidence interval or significance test for the difference between models is inferred from their separate intervals.

**Table 4:** Representative LLM and VLM applications in medical imaging.

Domain	Model or Method Class	Investigated Focus	Evaluation Issue and Representative Sources
General radiology	Foundation models and multimodal LLMs	Cross-modal retrieval, question answering, interpretation, and task transfer	Broad capability requires external, task-specific validation [39,40,41]
Chest radiography	Diagnostic and generative VLMs	Conversational interpretation, classification, and report generation	Common public datasets may not represent clinical deployment [42,43,44]
Specialized radiology	MRI, volumetric CT, and ultrasound VLMs	Tumour reasoning, report generation, and structured documentation	Evidence is smaller and less standardized than for chest radiography [45,46,47]
Adaptation methods	Parameter-efficient fine-tuning and reinforcement learning	Domain adaptation, segmentation, and medical reasoning	Adaptation can alter calibration and failure patterns [48,49,50]
Workflow tools	Agents and chatbots	Retrieval, tool use, case preparation, and user interaction	Sequential errors and automation bias require workflow testing [19,51,52]
Evaluation	Benchmarks and hallucination analysis	Factuality, grounding, robustness, and reasoning assessment	No single benchmark establishes clinical safety [53,54,55]

**Table 5:** Representative automated radiology report generation systems.

System	Imaging Domain	Principal Contribution	Important Evaluation Consideration
XRaySwinGen	Chest radiography	Hierarchical image encoding for report generation	Clinical factuality may not track lexical-overlap scores [57]
CT2Rep	Volumetric CT	Cross-modal modelling across three-dimensional examinations	Requires assessment of cross-slice and anatomical consistency [58]
MAIRA-2	Chest radiography	Grounded report generation linked to image regions	Grounding, omissions, and unsupported findings require separate assessment [17]
UIT-2Q2T	Mixed radiological images	BLIP-based medical-image captioning	A challenge result does not establish report quality in a clinical workflow [59]

**Table 6:** Representative directions in multimodal foundation models for radiology.

Model Direction	Key Characteristics	Representative Models	Investigated Applications	Sources
Generalist Medical Understanding	Unified multimodal reasoning and task adaptation	Lingshu, MedVersa, Citrus-V	Interpretation, retrieval, and image grounding	[18,62,63]
Oncology-Specific	Integration of radiology with clinical, pathology, or molecular data	HONeYBEE	Cancer classification, survival prediction, and patient retrieval	[64,65]
3D Imaging Specialized	Volumetric representation learning and image–report pairing	CT-CLIP, CT-CHAT	Classification, retrieval, report generation, and question answering	[66]
Generative Foundation Models	Multimodal data synthesis and conditional generation	XGeM	Conditional medical image–text generation	[67]
Multi-Disease Frameworks	Transfer evaluation across tasks	MerMED-FM	Multi-disease representation and transfer evaluation	[68]

**Table 7:** Representative condition-specific multimodal AI applications.

Condition	Integrated Modalities	Investigated Application	Modelling Focus	Sources
Pulmonary Infections	CT, radiological reports, laboratory tests	Pathogen identification and prognosis	Cross-modal feature integration	[31]
Tuberculosis	Radiography, clinical history	Disease classification and report generation	Vision–language alignment in a preprint study	[69]
Small Bowel Obstruction	CT, laboratory measurements, optional report text	Ischaemia assessment	Multicentre development and external testing	[70]
Ovarian Cancer	MRI, clinicopathology	Platinum sensitivity prediction	Habitat analysis integration	[33]

**Table 8:** Selected examples distinguishing predictive performance from clinical utility.

Study and Task	Inputs and Evaluation	Reported Finding	Interpretation and Boundary
Vanguri et al.: immunotherapy-response prediction [27]	Baseline CT, pathology and genomic features; 247-patient multimodal cohort with tenfold cross-validation. Additional cohorts tested unimodal features.	Integrated-model AUC 0.80 (95% CI 0.74–0.86), compared with PD-L1 score 0.73 and tumour mutational burden 0.61.	Supports incremental discrimination in the evaluated cohort. Separate unimodal validation does not establish external validation of the integrated model or benefit from model-guided treatment.
Shao et al.: pulmonary-infection classification [31]	CT and clinical information; retrospective evaluation including clinical-only and image-only comparisons, internal and external testing, and comparison with physicians.	Reported integrated-model AUC 0.910 (95% CI 0.904–0.916) internally and 0.887 (95% CI 0.867–0.909) externally.	Supports predictive performance across the tested settings. These results do not themselves demonstrate improved prescribing, clinician decisions, or patient outcomes from deployment.
MAIRA-2: grounded chest-radiograph reporting [17]	Radiographs and reporting context; report-generation benchmarks and sentence-level factuality and grounding evaluation using RadFact.	Reported improvements in benchmark report generation and introduced image-linked findings.	Supports technical reporting capability under the evaluated conditions. Benchmark and grounding results alone do not establish reduced verification burden or safer final reports in prospective practice.

**Table 9:** Proposed stages for evaluating multimodal radiology AI before and after clinical deployment.

Stage	Main Evaluation	Evidence Required to Extend the Clinical Claim
1. Retrospective development and internal validation	Separate patients and time points appropriately; audit information leakage; compare relevant unimodal and multimodal models using matched data. Examine missing inputs, calibration, and clinically significant errors.	Demonstrate the intended technical performance on held-out data and justify the contribution and timing of each modality. Internal success supports further testing.
2. External validation	Test the complete, fixed system on independent institutions or populations with different acquisition and documentation practices. Assess subgroup performance and realistic missing or conflicting inputs.	Establish generalizability within the tested settings. Report any recalibration separately; reserve independent data to evaluate subsequent adaptation.
3. Reader and workflow studies	Compare radiologists with and without assistance using representative cases and suitable randomization or counterbalancing. Measure decision accuracy, error detection, final-report quality, verification time, and inappropriate reliance.	Show whether the combined clinician–AI process provides benefit under the evaluated conditions. Controlled reader studies do not establish patient benefit in routine care.
4. Prospective clinical evaluation	Begin with feasibility and safety evaluation in the intended workflow; then conduct an appropriately controlled impact study, randomized where suitable. Prespecify clinical or workflow outcomes, adverse events, and stopping rules.	Demonstrate the claimed benefit of using the system relative to usual care. Silent prospective testing can assess technical performance but cannot establish the effect of acting on AI output.
5. Post-deployment monitoring	Audit performance as outcomes become available; track input drift, missingness, subgroup errors, overrides, incidents, and model or interface changes. Assign responsibility for review and corrective action.	Maintain acceptable performance over time, with triggers for investigation, restriction, or withdrawal. Material changes require renewed evaluation before extending prior claims to an updated system.

## Data Availability

No new data were created or analyzed in this study. Data sharing is not applicable to this article.

## Abbreviations

The following abbreviations are used in this manuscript:

AI	Artificial intelligence
ANN	Artificial neural network
CNN	Convolutional neural network
EHR	Electronic health record
LLM	Large language model
NLP	Natural language processing
VLM	Vision–language model
XAI	Explainable artificial intelligence

## Appendix A. Search Strategy and Data-Charting Framework

### Appendix A.1. Deduplication Methodology

Records from all databases were imported into Mendeley reference management software. Deduplication was performed using automatic duplicate detection based on title, author names, publication year, and DOI when available. Manual review was used to resolve near-duplicate records and preprint/published-article duplicates.

### Appendix A.2. Search Strategy

Searches combined terms for multimodal artificial intelligence, radiology, medical imaging, clinical text, radiology reports, VLMs, LLMs, and foundation models. Searches were adapted to each database syntax. The reviewed publications included original research, reviews, and commentaries; reference lists also supported citation tracking. The selection principles and screening counts are reported in Section 2.3; Figure 1 summarizes the documented selection process. The reported total counts publications, not independent empirical studies.

**Table A1:** Database-specific search strategy summary.

Database/Source	Core Search String or Adaptation	Notes
PubMed	(“multimodal artificial intelligence” OR “multimodal AI” OR “vision-language model” OR “large language model” OR “foundation model”) AND (radiology OR “medical imaging” OR “diagnostic imaging”) AND (“clinical text” OR “radiology report” OR “clinical notes” OR EHR)	MeSH and title/abstract terms were combined where available.
IEEE Xplore/ACM Digital Library	(“multimodal AI” OR “vision-language” OR “cross-modal” OR “foundation model”) AND (radiology OR “medical imaging”) AND (text OR report OR “clinical notes”)	Used to capture technical conference and engineering literature.
Web of Science/Scopus	TITLE-ABS-KEY terms for multimodal AI, radiology or medical imaging, and clinical text or reports	Used for interdisciplinary coverage and citation tracking.
arXiv	(“multimodal” OR “vision-language” OR “foundation model” OR “large language model”) AND (radiology OR “medical imaging”)	Used to capture emerging preprint literature.
Google Scholar	Targeted searches combining multimodal AI, radiology, clinical text, reports, VLM, LLM, and foundation model	Used supplementally; results were screened in relevance order.

### Appendix A.3. Data-Charting Framework

The following Table lists the variables used to chart reviewed publications, where applicable, and organize the descriptive synthesis.

**Table A2:** Literature-charting variables used for the narrative review.

Data-Charting Variable	Details Extracted
Bibliographic details	Author, year, publication type, journal or conference, and peer-reviewed or preprint status
Study context	Country or region, clinical domain, and radiology subspecialty
Data modalities	Imaging modality, text source, and structured or unstructured clinical context
Technical approach	Model architecture, fusion strategy, pretraining approach, and fine-tuning strategy
Clinical task	Diagnosis, prognosis, report generation, retrieval, question answering, segmentation, and workflow support
Evaluation	Datasets, metrics, comparator models, external validation, and prospective validation
Translation and ethics	Interpretability, fairness, privacy, hallucination, workflow integration, and regulatory considerations

### Appendix A.4. Search Update

If this review is updated in the future, the same search strategies should be re-run from 1 May 2026 onward to identify newly published studies. Search syntax may require modification if database platforms change or if new MeSH terms and keywords emerge.

## Appendix B. Complete Inventory of Reviewed Publications

Table A3 lists all 111 reviewed publications. Each inventory ID links to a numbered reference with full bibliographic details. The corpus includes original research and contextual publications, including reviews and commentaries; inclusion is not a quality rating. Gaps in the identifiers correspond to Wood and Savova et al., both background references outside the corpus. Nine references added during revision are also outside the 111. Names and years follow the cited bibliography; related preprint and journal versions are counted once.

**Table A3:** Complete cited inventory of the 111 reviewed publications. Read each row from left to right.

ID	Publication	ID	Publication
R001	McBee et al. (2018) [2]	R003	Benani et al. (2025) [4]
R005	Summaira et al. (2021) [6]	R006	Chen et al. (2023) [8]
R007	Nensa (2025) [9]	R008	Pawłowski et al. (2023) [7]
R009	Band et al. (2023) [10]	R010	Li et al. (2022) [11]
R011	Ranschaert et al. (2021) [13]	R012	Khan et al. (2026) [12]
R013	Page et al. (2021) [14]	R014	Shetty et al. (2023) [20]
R015	Hsieh et al. (2023) [21]	R016	Khader et al. (2023) [23]
R017	Yao et al. (2024) [24]	R018	Khader et al. (2023) [25]
R019	Zhang et al. (2025) [16]	R020	Buckley et al. (2023) [28]
R021	Huang et al. (2021) [15]	R022	Zhou et al. (2021) [22]
R023	Nakach et al. (2024) [26]	R024	Vanguri et al. (2022) [27]
R025	Mohsen et al. (2022) [29]	R026	Oncu et al. (2025) [30]
R027	Alaei et al. (2025) [79]	R028	Milosevic et al. (2024) [34]
R029	Wang et al. (2024) [35]	R030	Zhang et al. (2025) [36]
R031	Can et al. (2025) [37]	R032	Abdullakutty et al. (2024) [80]
R033	Yu et al. (2025) [81]	R034	Maqsood et al. (2026) [32]
R035	Shao et al. (2024) [31]	R036	Ketabi et al. (2025) [38]
R037	Dąbrowicki et al. (2025) [82]	R038	Huang et al. (2025) [83]
R039	Bi et al. (2025) [33]	R040	Huang et al. (2024) [84]
R041	Yildirim et al. (2024) [39]	R042	Bradshaw et al. (2025) [85]
R043	Gupta (2026) [86]	R044	Nakaura et al. (2024) [87]
R045	Kalpelbe et al. (2025) [88]	R046	Paschali et al. (2025) [89]
R047	Gu et al. (2026) [90]	R048	Gao et al. (2025) [91]
R049	Zhu et al. (2024) [40]	R050	Hu et al. (2026) [92]
R051	Li et al. (2023) [93]	R052	Wang et al. (2026) [94]
R053	Yan et al. (2023) [95]	R054	Huang et al. (2025) [96]
R055	Liu et al. (2023) [97]	R056	Strotzer et al. (2024) [41]
R057	Das et al. (2025) [54]	R058	Panagoulias et al. (2024) [98]
R059	Moon et al. (2022) [99]	R060	Qiu et al. (2024) [100]
R061	Lu et al. (2025) [101]	R062	Nath et al. (2025) [102]
R063	Dhinagar et al. (2025) [103]	R064	Lee et al. (2025) [42]
R065	Park et al. (2025) [43]	R066	Alkhaldi et al. (2024) [104]
R067	Davis et al. (2025) [105]	R068	Sharma et al. (2024) [106]
R069	Zhang et al. (2025) [44]	R070	Ng et al. (2025) [55]
R071	Zhan et al. (2026) [107]	R072	Li et al. (2025) [108]
R073	Safari et al. (2026) [45]	R074	Li et al. (2025) [46]
R075	Ge et al. (2025) [47]	R076	Khanal et al. (2026) [109]
R077	Basiri et al. (2025) [110]	R078	Candito et al. (2025) [51]
R079	Chen et al. (2025) [19]	R080	He et al. (2024) [48]
R081	Huang et al. (2025) [49]	R082	Su et al. (2025) [50]
R083	Liu et al. (2025) [53]	R084	Wang et al. (2024) [111]
R085	Bhayana (2024) [52]	R086	Agarwal et al. (2025) [112]
R087	Ghosh et al. (2024) [113]	R088	Rao et al. (2025) [114]
R089	Yang et al. (2024) [115]	R090	Deria et al. (2026) [56]
R091	Singh et al. (2026) [116]	R092	Niu et al. (2024) [117]
R093	Veras Magalhães et al. (2024) [57]	R094	Hamamci et al. (2024) [58]
R095	Bannur et al. (2024) [17]	R096	Phan et al. (2024) [59]
R097	Salmè et al. (2025) [60]	R098	LASA Team et al. (2025) [62]
R099	Zhou et al. (2026) [18]	R100	Wang et al. (2025) [63]
R101	Menon et al. (2026) [65]	R102	Tripathi et al. (2025) [64]
R103	Hamamci et al. (2026) [66]	R104	Molino et al. (2026) [67]
R105	Zhou et al. (2025) [68]	R106	Muneer et al. (2025) [118]
R107	Bian et al. (2025) [119]	R108	Raposo (2026) [120]
R109	Petridis et al. (2026) [121]	R110	Shastry et al. (2025) [69]
R111	Lu et al. (2025) [122]	R112	Vanderbecq et al. (2026) [70]
R113	Mehrtabar et al. (2025) [71]		
